# Different contributions of efferent and reafferent feedback to sensorimotor temporal recalibration

**DOI:** 10.1038/s41598-021-02016-5

**Published:** 2021-11-19

**Authors:** Belkis Ezgi Arikan, Bianca M. van Kemenade, Katja Fiehler, Tilo Kircher, Knut Drewing, Benjamin Straube

**Affiliations:** 1 Experimental Psychology, Justus Liebig University Giessen, Otto-Behaghel Strasse 10F, 35394 Giessen, Germany; 2 Center for Mind, Brain and Behavior (CMBB), Philipps University Marburg and Justus Liebig University Giessen, Hans-Meerwein-Strasse 6, 35032 Marburg, Germany; 3 Institute of Neuroscience and Psychology, University of Glasgow, 62 Hillhead Street, Glasgow, G12 8QB UK; 4 Department of Psychiatry and Psychotherapy, Philipps University Marburg, Rudolf-Bultmann-Strasse 8, 35039 Marburg, Germany

**Keywords:** Human behaviour, Perception, Sensorimotor processing

## Abstract

Adaptation to delays between actions and sensory feedback is important for efficiently interacting with our environment. Adaptation may rely on predictions of action-feedback pairing (motor-sensory component), or predictions of tactile-proprioceptive sensation from the action and sensory feedback of the action (inter-sensory component). Reliability of temporal information might differ across sensory feedback modalities (e.g. auditory or visual), which in turn influences adaptation. Here, we investigated the role of motor-sensory and inter-sensory components on sensorimotor temporal recalibration for motor-auditory (button press-tone) and motor-visual (button press-Gabor patch) events. In the adaptation phase of the experiment, action-feedback pairs were presented with systematic temporal delays (0 ms or 150 ms). In the subsequent test phase, audio/visual feedback of the action were presented with variable delays. The participants were then asked whether they detected a delay. To disentangle motor-sensory from inter-sensory component, we varied movements (active button press or passive depression of button) at adaptation and test. Our results suggest that motor-auditory recalibration is mainly driven by the motor-sensory component, whereas motor-visual recalibration is mainly driven by the inter-sensory component. Recalibration transferred from vision to audition, but not from audition to vision. These results indicate that motor-sensory and inter-sensory components contribute to recalibration in a modality-dependent manner.

## Introduction

Perceiving sensory events almost always involves dealing with temporal discrepancies. Discrepancies may result from temporal differences in neural transduction^1,2^, developmental changes^3^, or physical characteristics of a given sensory input^4^. Yet, humans are highly efficient in compensating for these discrepancies. For example, temporal misalignment between our actions and their sensory feedback resulting from any or a combination of the above-mentioned factors can be accommodated. This is known as sensorimotor temporal recalibration. Temporal adjustment of sensorimotor events has been demonstrated for actions leading to a specific sensory feedback^5–9^ as well as for actions leading to feedback from multiple senses (i.e., audiotactile feedback when knocking on a door)^10–12^. Recalibration manifests itself in the adjustment of timing of either the initial (action) or the resulting (sensory feedback) event. As a consequence, the action and feedback are *perceived* in congruence with each other. In an extreme case, recalibration of time between an action and its feedback may lead to the illusory perception of feedback occurring *before* the action. In a seminal study, Stetson et al.^8^ had participants adapt to delays of 135 ms between voluntary button press-flash pairs. When this delay was eliminated after adaptation, participants experienced an illusory perception of the flash preceding the button press, although its physical timing was not preceding the action (see also^5,13^). Sensorimotor temporal recalibration aids not only in the binding of sensory and motor events that belong together, but also in attributing control (agency) over the events we generate^14–16^. This suggests that temporal recalibration is specific to the events that are causally-related. However, voluntary actions seem to provide us with an additional advantage in recalibrating temporal perception^17^. Actions can trigger an internal forward model which predicts the sensory feedback of the action based on the efference copy of the motor command^18–20^. Such predictive processing may lead to stronger adaptation of motor-sensory event pairs relative to purely sensory event pairs. Indeed, it has been consistently demonstrated that actions provide a temporal window into which binding of sensory events can be facilitated^11,17,21,22^.

Temporal discrepancies between actions and sensory feedback result in misalignment of two components: a motor-sensory component (misalignment between the action and its sensory feedback), and an inter-sensory component (misalignment between cross-modal sensory inputs); one or both needs to be recalibrated^9,23^. For example, whenever we click a link to a website, some amount of time is required to load and display the page on our computer screen. The *perceived* interval between these events may be due to temporal misalignment between the click and the appearance of the website (motor-sensory component), or between the tactile-proprioceptive feedback arising from the click and the appearance of the website (inter-sensory component). In order to attribute causality between the click and the appearance of the website, the perceived timing of these events are adjusted.

How do motor-sensory and inter-sensory components contribute to sensorimotor temporal recalibration? A number of studies have addressed the differential contributions of these components by investigating how temporal or spatial perturbations to sensorimotor events are dynamically adjusted. The perturbation, identified as error, violates the predicted (learned) relationship between the sensorimotor event pair, and can either be random or systematic^24,25^. Adaptation maximizes the accuracy of predictions by minimizing systematic errors^24^. The degree to which these errors contribute to adaptation depends on the reliability of the error; i.e., the more reliable estimate with minimum variance receives a higher weight, and therefore, contributes more to adaptation^24–26^. Other studies investigating the relative contributions of motor-sensory and inter-sensory components on adaptation aimed to disentangle predictions based on efference copy from reafferent feedback, and test which component accounts mostly for recalibration effects. Comparing adaptation during voluntary button presses with a *passive* condition in which the button moved the finger, Stetson et al.^8^ found larger temporal recalibration effects for voluntary button presses triggering flashes, and smaller recalibration effects for the passive button presses, indicating the importance of action intention (in which efference copy is present) on sensorimotor recalibration. In another study, Arnold et al.^23^ examined the role of action intentions on sensorimotor temporal recalibration using ballistic reaches with short or longer extent before a voluntary button press triggered a tone. By manipulating the time between the intention to act and the auditory feedback of the action, the authors were able to investigate whether the intention to act or the sensation of having acted drives sensorimotor temporal recalibration. They found that the temporal relationship between tactile signals associated with completion of the action and the auditory feedback of the action determines recalibration rather than the action itself, highlighting the role of the inter-sensory component over the motor-sensory component.

Apart from contributions of motor-sensory and inter-sensory components, the sensory modality of the action feedback (e.g., auditory or visual) may also impact temporal recalibration^27–29^. In general, audition has superior temporal resolution compared to the other senses^30,31^. This has also been observed when sensory modalities are processed together. For example, audio-tactile events have been found to have higher temporal resolution than visuo-tactile or audio-visual events^32^, suggesting higher reliability for auditory events in the temporal domain. A number of studies addressing the role of sensory feedback modality on recalibration tested whether recalibration transfers to another modality. Accordingly, after adapting to a delay between a button press and a visual feedback, the visual feedback would be replaced by an auditory feedback^6,9,27^. Transfer of recalibration from the visual to the auditory feedback (and vice versa) indicates a supramodal (modality a-specific) mechanism which is not affected by the sensory feedback modality, whereas absence of transfer underlies a modality-specific mechanism^6,9,27^. Sugano et al.^27^ found transfer of recalibration from vision to audition, but not the other way around. This supports the presence of a modality-specific mechanism (but see^6,9^ for supramodal recalibration effects). The transfer effects may also provide information on which event is shifted in time: a modality-specific transfer effect might indicate that the perceived timing of the initial (motor) event has shifted in time towards the resulting event (sensory feedback)^27^. Such differential transfer effects allow one to test whether different sensory modalities interact differently with motor-sensory and inter-sensory components in influencing sensorimotor temporal recalibration.

Existing evidence on sensorimotor temporal recalibration suggests that motor-sensory and inter-sensory components are likely modulated by cross-modal interactions. Importantly, studies on modality-specific recalibration have not disentangled how efferent (corresponding to the motor-sensory component) and reafferent (corresponding to the inter-sensory component) feedback contribute to temporal recalibration^6,9,27,29^. On the other hand, studies investigating the contribution of these components have not addressed possible modality-specific effects^8,23^. To our knowledge, no study has explored the contributions of motor-sensory and inter-sensory components (disentangling efference copy from reafferent feedback) on sensorimotor temporal recalibration while addressing possible cross-modal interactions. Our aim in the present study is to investigate sensorimotor temporal recalibration for motor-auditory and motor-visual events by disentangling the influence of motor-sensory component and inter-sensory component. To this end, we used a recalibration paradigm in which systematic temporal delays between actions and their sensory feedback were introduced (adaptation phase). To assess temporal recalibration, we tested participants’ perception for variable delays inserted between action-feedback events (test phase). Crucially, we used active and passive movements at adaptation and test phases to disentangle motor-sensory and inter-sensory components of recalibration. In the condition where button presses at adaptation and test are both passive (adapt passive, test-passive), a *purely* inter-sensory adaptation between the tactile-proprioceptive and the auditory or visual feedback is expected. Comparing this passive condition with active (voluntary) button presses at adaptation and test (adapt-active, test-active) may aid in understanding the relation between sensorimotor and purely sensory recalibration, and possible recalibration differences as a function of accompanying sensory feedback^6,8,9^. In the third condition, efferent feedback is not present at adaptation, but is present at test (adapt-passive, test-active). Because efferent feedback cannot be adapted, any adaptation effect we observe under this condition should result from the inter-sensory component. Examining differences between the adapt-passive, test-active and the adapt-active, test-active condition (in which the efferent feedback can be adapted) may hence address the specific contribution of the two components.

Possible mechanisms underlying sensorimotor temporal recalibration involve a shift of one of the events in time towards the other (e.g., shift in the timing of the sensory feedback towards the motor event), and a widening of the temporal window of integration between the events^33^. These mechanisms are not mutually exclusive, and can all contribute to recalibration^33^. In the present study, we tested for these possibilities by comparing detection thresholds and just-noticeable differences (JNDs) across adaptation delays. A systematic increase in detection thresholds or JNDs in the 150 ms delay as opposed to 0 ms delay in a specific condition would correspond to recalibration. Drawing from previous work on sensorimotor recalibration^8,9,23–26^ and on inter-sensory recalibration^34–36^, we argue that sensorimotor temporal recalibration results from a combination of motor-sensory and inter-sensory components modulated by the reliability of the action feedback in perceiving time. We hypothesized that, if the motor-sensory component is more reliable due to predictions based on efference copy^18–20^, which enhances the temporal organization of events^10,11,14,17,21^, then larger adaptation effects should be present for sensorimotor recalibration (adapt-active, test-active condition) than for purely sensory recalibration (adapt-passive, test-passive condition). We further hypothesized that sensorimotor temporal recalibration would result from a combination of motor-sensory component (corresponding to predictions based on efference copy) and inter-sensory component (corresponding to reafferent feedback), and that the contributions of each component depend on their reliability^24–26^. If efferent feedback is more reliable than reafferent feedback in terms of recalibrating temporal discrepancies in action-feedback pairs^8^, we expect lower impact of inter-sensory discrepancies, and higher impact of motor-sensory discrepancies. This would suggest that negligible or no recalibration would occur in the adapt-passive, test-active condition compared to the adapt-active, test-active condition. Alternatively, if reafferent feedback is more reliable for recalibration^23^, then similar adaptation profiles should be evident in the adapt-active, test-active and adapt-passive, test-active conditions, as both conditions contain reafferent feedback (inter-sensory component) in the adaptation phase. Differences across adapted sensory modalities, if found, would point to a change in the relative influence of the motor-sensory or the inter-sensory component depending on the adapted sensory modality.

Finally, we hypothesized that a transfer from an adapted to a non-adapted sensory modality would indicate supramodal recalibration, which is not influenced by the sensory feedback of the action^6,9^, whereas lack of transfer would suggest a modality-specific influence on recalibration^27,28,37^. Importantly, the existence of transfer, along with differences in recalibration across adapt-active, test-active and adapt-passive, test-active conditions, would provide insight into which component contributes more in recalibrating action-feedback discrepancies. For example, if the adapted sensory modality is shifted in time, indicating a shift of the sensory feedback either towards the motor-sensory or the inter-sensory component (or both), then there should be no cross-modal transfer. Likewise, transfer of recalibration from vision to audition in the adapt-active, test-active but not in the adapt-passive, test-active condition would indicate that the motor-sensory component is shifted in time.

## Results

**Figure 1:**
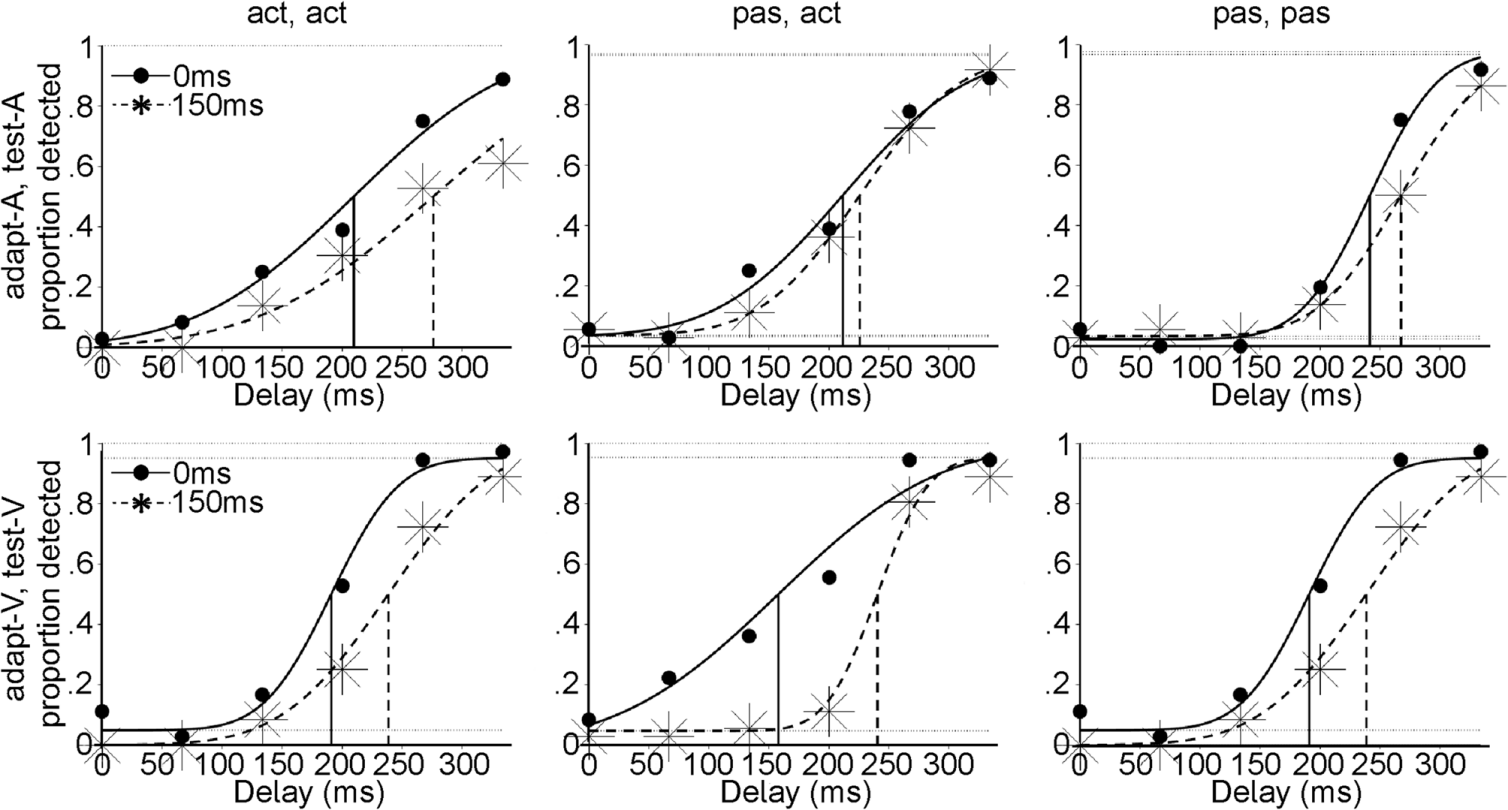
Plots showing detection responses and psychometric function fits for each condition from a representative participant. Filled circles and stars show proportion of detected responses as a function of test delay in the 0 ms and 150 ms adaptation delay conditions, respectively. A shift in detection thresholds from 0 to 150 ms delay indicates adaptation.

Table 1 shows group-level threshold and JND estimates for each condition. Figure 1 depicts detection responses as a function of delay for each condition from a representative participant. Overall, detection thresholds were higher in the 150 ms condition than in the 0 ms condition, indicating recalibration.

**Table 1:** Mean (standard error of mean, s.e.m.) thresholds and JNDs for each condition.

	Adapt-active, test-active	Adapt-passive, test-active	Adapt-passive, test-passive
**Threshold (ms)**			
Adapt-A, test-A			
0 ms	202.53 (19.45)	226.36 (20.25)	241.83 (13.78)
150 ms	229.63 (19.83)	230.21 (18.02)	254.44 (11.82)
Adapt-V, test-V			
0 ms	235.59 (19.98)	232.87 (21.02)	236.96 (13.90)
150 ms	261.60 (17.88)	263.82 (18.63)	258.23 (14.53)
Adapt-A, test-V			
0 ms	240.06 (21.93)	243.10 (23.11)	241.31 (16.84)
150 ms	248.32 (16.62)	252.56 (16.42)	252.27 (19.76)
Adapt- V, test-A			
0 ms	225.66 (19.37)	235.18 (19.07)	243.57 (20.67)
150 ms	248.10 (23.58)	237.83 (23.23)	263.55 (15.53)
**JND (ms)**			
Adapt-A, test-A			
0 ms	75.84 (9.97)	73.50 (7.07)	71.02 (10.78)
150 ms	65.80 (8.39)	68.94 (4.81)	70.67 (12.61)
Adapt-V, test-V			
0 ms	74.24 (9.44)	81.82 (13.12)	72.09 (11.48)
150 ms	62.30 (5.05)	66.53 (5.95)	67.62 (6.50)
Adapt-A, test-V			
0 ms	71.06 (8.59)	72.06 (11.87)	79.57 (13.20)
150 ms	72.75 (11.91)	80.88 (9.64)	77.96 (11.49)
Adapt-V, test-A			
0 ms	68.88 (10.47)	76.45 (10.75)	74.19 (9.82)
150 ms	79.03 (9.54)	72.62 (8.75)	78.58 (11.83)

### The impact of motor-sensory and inter-sensory components on sensorimotor temporal recalibration

**Figure 2:**
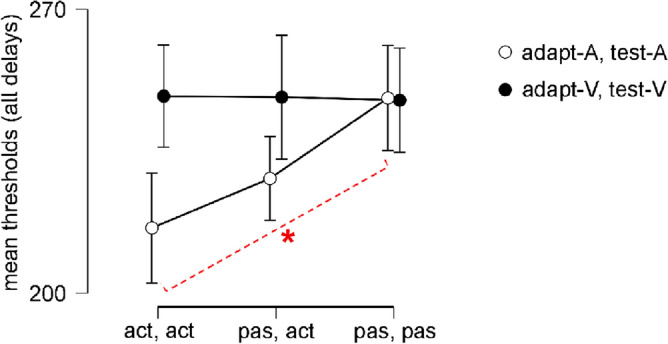
Line plots showing detection thresholds in the adapt-active, test-active (act, act), adapt-passive, test-active (pas, act) and adapt-passive, test-passive (pas, pas) conditions averaged across adaptation delays. Bold asterisk shows significant differences between conditions. Error bars represent the standard error of the mean (s.e.m.).

**Figure 3:**
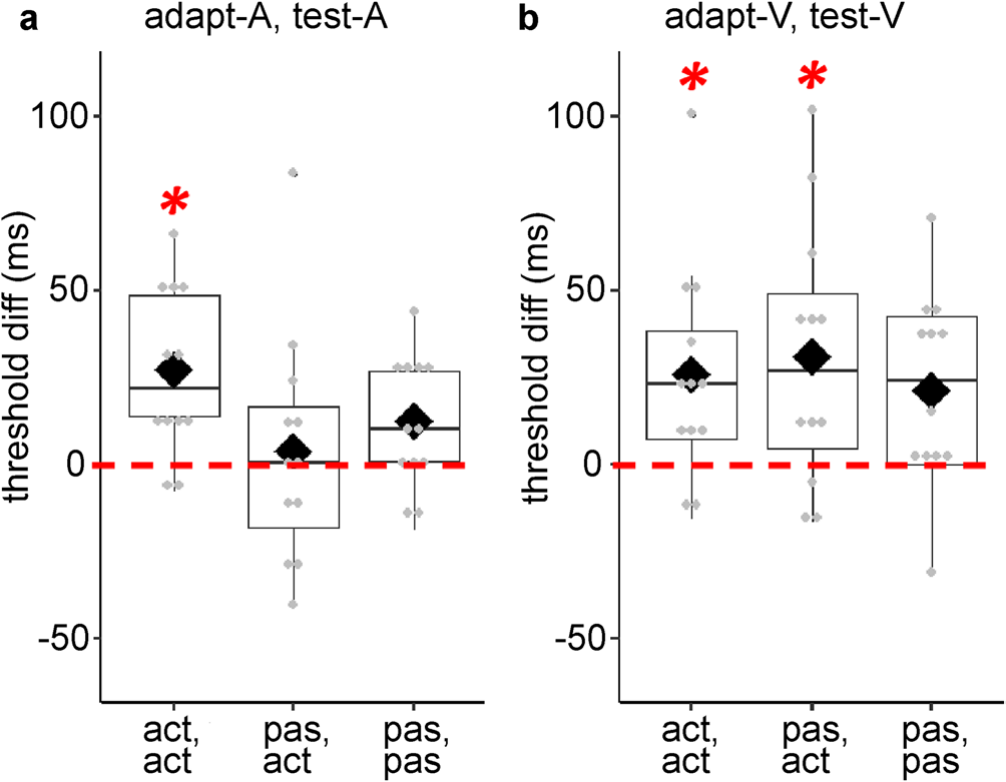
Boxplots with individual data points showing significant effect of adaptation delay (threshold differences between 150 and 0 ms delay conditions) as a function of *adaptation, test mode* in (**a**) the adapt-A, test-A condition, and (**b**) the adapt-V, test-V condition. The dashed line depicts 0 difference. Diamonds and solid lines show the mean and the median values of the data, respectively. Asterisks show significant effects.

The 3 (*adaptation, test mode*: adapt-active, test-active vs. adapt-passive, test-active vs. adapt-passive, test-passive) × 2 (*adaptation, test modality*: adapt-A, test-A vs. adapt-V, test-V) × 2 (*adaptation delay*: 0 ms vs. 150 ms) repeated measures ANOVA on thresholds revealed a significant main effect of *adaptation delay* (see Table 2). Detection thresholds in the 0 ms delay condition (mean = 229.36, s.e.m. = 17.02) were significantly smaller than those in the 150 ms delay condition (mean = 249.65, s.e.m. = 15.27). There was also a two-way interaction between *adaptation, test mode* and *adaptation, test modality* (see Table 2; cf. Fig. 2), and a three-way interaction between *adaptation, test mode*; *adaptation, test modality* and *adaptation delay* (see Table 2; cf. Fig. 3). For the two-way interaction, post-hoc comparisons were performed for *adaptation, test mode* in the adapt-A, test-A condition given similar mean thresholds across movements for the adapt-V, test-V conditions. The planned post-hoc comparisons showed that mean detection thresholds (independent of *adaptation delay*) were significantly smaller in the adapt-active, test-active (mean = 216.08, s.e.m. = 19.34) than in the adapt-passive, test-passive (mean = 248.14, s.e.m. = 12.55) condition for the adapt-A, test-A modality; *t*(11) = − 4.30, *p* = 0.002, *d* = 1.44 (see Fig. 2). All other effects were non-significant (see Table 2).

**Table 2:** Repeated-measures ANOVA results on detection thresholds within modalities.

Effects	df	F	*p*	*ƞ*_*p*_^2^
Adaptation, test mode	2, 22	2.72	0.088^tt^	0.20
Adaptation, test modality	1, 11	3.58	0.085^tt^	0.25
Adaptation delay	1, 11	15.50	0.001*^ot^	0.59
Adaptation, test mode × Adaptation, test modality	2, 22	13.51	< 0.001*^tt^	0.55
Adaptation, test mode × adaptation delay	2, 22	2.32	0.122^tt^	0.17
Adaptation, test modality × adaptation delay	1, 11	1.18	0.301^tt^	0.10
Adaptation, test mode × adaptation, test modality × adaptation delay	2, 22	3.77	0.039*^tt^	0.26

Bold asterisks indicate significant effects. ‘^ot^’ indicate one-tailed test values whereas ‘^tt^’ indicate two-tailed test values. df, degrees of freedom; F, F value, *p*, *p* value; *ƞ*_*p*_^2^ partial eta-squared.

For the three-way interaction, we conducted planned comparisons testing the presence of adaptation in each condition, namely the difference between the adaptation delays for each *adaptation, test mode* and *adaptation, test modality*. For each participant, we subtracted the detection thresholds in the 0 ms delay condition from the 150 ms delay condition separately for *adaptation, test mode* and *modality*, and tested the differences (threshold_Diff_) against 0 with one-sample t-tests. As the expected direction of the effect was specific (larger detection thresholds for the 150 ms delay compared to 0 ms delay), we used one-tailed t-tests, corrected for multiple comparisons. The results showed significantly different threshold_Diff_ values for the following conditions: adapt-active-A, test-active-A; adapt-active-V, test-active-V; adapt-passive-V, test-active-V (see Table 3 and Fig. 3).

**Table 3:** One sample t-test results on threshold differences (threshold_Diff_) between 150 and 0 ms adaptation delays, indication recalibration within modalities.

Threshold_diff_ (150–0 ms)	t	df	*p*	*d*
Adapt-active-A, test-active-A	3.96	11	0.001*^ot^	1.14
Adapt-passive-A, test-active-A	0.40	11	0.349^ot^	0.12
Adapt-passive-A, test-passive-A	2.33	11	0.019^ot^	0.67
Adapt-active-V, test-active-V	2.88	11	0.0075*^ot^	0.83
Adapt-passive-V, test-active-V	2.85	11	0.0079*^ot^	0.82
Adapt-passive-V, test-passive-V	2.62	11	0.0119^ot^	0.76

Bold asterisks indicate significant effects. ‘^ot^’ indicate one-tailed test values. t, value; df, degrees of freedom; *d*, Cohen’s *d*. All tests were corrected for multiple comparisons.

In order to assess the relative impact of the inter-sensory component compared to the motor-sensory component for motor-visual events, we conducted a paired samples t-test on threshold differences between adapt-active, test-active and adapt-passive, test-active conditions when *adaptation, test modality* was visual. There were no significant differences between the conditions, *t*(11) = 0.78, *p* = 0.45, *d* = 0.23. This suggests that the inclusion of the motor-sensory component at adaptation did not significantly contribute to the shift in detection thresholds for motor-visual events.

The 3 (*adaptation, test mode*: adapt-active, test-active vs. adapt-passive, test-active vs. adapt-passive, test-passive) × 2 (*adaptation, test modality*: adapt-A, test-A vs. adapt-V, test-V) × 2 (*adaptation delay*: 0 ms vs. 150 ms) repeated measures ANOVA on JNDs resulted in no significant differences across conditions (see Table 4). Together, the results suggest a shift in the detection thresholds as a function of adaptation delay for the visual modality when an active movement was present at test. For the auditory modality, recalibration is present in the adapt-active, test-active condition. JNDs across adaptation delays were similar, suggesting no differences in the temporal window of integration for event pairs as a function of delay at adaptation.

**Table 4:** Repeated-measures ANOVA results on JNDs within modalities.

Effects	df	F	*p*	*ƞ*_*p*_^2^
Adaptation, test mode	2, 22	0.31	0.74^tt^	0.03
Adaptation, test modality	1, 11	0.003	0.96^tt^	< 0.01
Adaptation delay	1, 11	2.81	0.06^ot^	0.20
Adaptation, test mode × adaptation, test modality	2, 22	0.20	0.82^tt^	0.02
Adaptation, test mode × adaptation delay	2, 22	0.67	0.52^tt^	0.06
Adaptation, test modality × adaptation delay	1, 11	0.46	0.51^tt^	0.04
Adaptation, test mode × adaptation, test modality × adaptation delay	2, 22	0.37	0.69^tt^	0.03

‘^ot^’ indicate one-tailed test values whereas ‘^tt^’ indicate two-tailed test values. df, degrees of freedom; F, F value, *p*, *p* value; *ƞ*_*p*_^2^ partial eta-squared.

### Cross-modal transfer of recalibration

**Figure 4:**
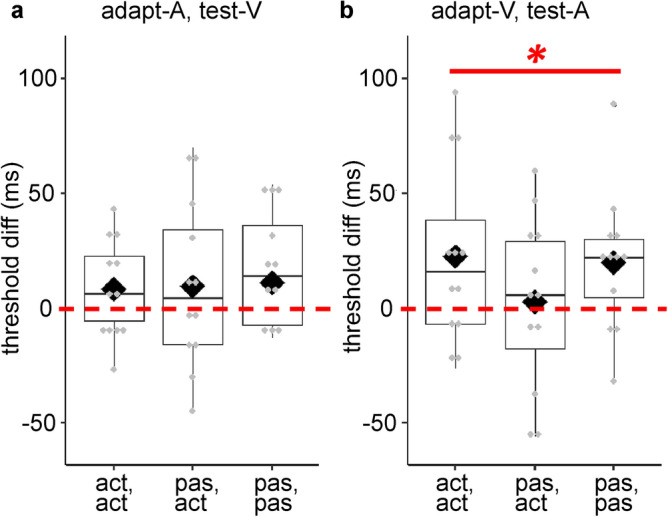
Boxplots with individual data points showing threshold differences between the 150 ms and 0 ms delay conditions as a function of *adaptation, test mode* in (**a**) the adapt-A, test-V, and (**b**) the adapt-V, test-A condition. Bold asterisk shows significant main effect of *adaptation delay*. The dashed line depicts 0 difference. Diamonds and solid lines show the mean and the median of the points, respectively.

Figure 4 shows threshold differences across adaptation delays for each cross-modal condition.

Results of the initial analysis suggest a shift in the detection thresholds with adaptation delays (confirming recalibration), and differences in recalibration across sensory modalities. We therefore examined transfer of recalibration separately for each *adaptation, test modality,* including only those conditions that produced significant within-modality adaptation (see Supplementary Materials for an overview of threshold differences across adaptation delays for each cross-modal condition, and analyses including all *adaptation, test modalities* even when significant within-modality recalibration is lacking).

For the adapt-A condition, the 2 (*adaptation, test modality*: adapt-A, test-A vs. adapt-A, test-V) × 2 (*adaptation delay*: 0 ms vs. 150 ms) repeated measures ANOVA on thresholds revealed a main effect of *adaptation, test modality*, a main effect of *adaptation delay*, and an interaction (see Table 5). A one sample t-test on the threshold_Diff_ for adapt-active-A, test-active-V condition was not significant; t(11) = 1.36, *p* = 0.10 (one-sided), *d* = 0.39. The 2 (*adaptation, test modality*: adapt-A, test-A vs. adapt-A, test-V) × 2 (*adaptation delay*: 0 ms vs. 150 ms) repeated measures ANOVA on JNDs for the adapt-active, test-active revealed no significant main effects or interaction effects (see Table 5).

**Table 5:** Repeated-measures ANOVA results on detection thresholds and JNDs assessing transfer effects for adapt-A conditions.

Effects	df	F	*p*	*ƞ*_*p*_^2^
**Detection thresholds**				
Adaptation, test modality	1, 11	10.33	0.008*^tt^	0.48
Adaptation delay	1, 11	11.64	0.003*^ot^	0.51
Adaptation, test modality × adaptation delay	1, 11	5.84	0.03*^tt^	0.35
**JNDs**				
Adaptation, test modality	1, 11	0.04	0.85^tt^	0.003
Adaptation delay	1, 11	1.70	0.11^ot^	0.13
Adaptation, test modality × adaptation delay	1, 11	2.18	0.17^tt^	0.17

Bold asterisks indicate significant effects. ‘^ot^’ indicate one-tailed test values whereas ‘^tt^’ indicate two-tailed test values. df, degrees of freedom; F, F value, *p*, *p* value; *ƞ*_*p*_^2^ partial eta-squared.

The 2 (*adaptation, test mode*: adapt-active, test-active vs. adapt-passive, test-active) × 2 (*adaptation, test modality*: adapt-V, test-V vs. adapt-V, test-A) × 2 (*adaptation delay*: 0 ms vs. 150 ms) repeated measures ANOVA on thresholds for adapt-V conditions resulted in a main effect of *adaptation delay* (see Fig. 4b and Table 6), indicating an overall shift in the detection thresholds between 0 ms vs. 150 ms delays across all conditions. The 2 (*adaptation, test mode*: adapt-active, test-active vs. adapt-passive, test-active) × 2 (*adaptation, test modality*: adapt-V, test-V vs. adapt-V, test-A) × 2 (*adaptation delay*: 0 ms vs. 150 ms) repeated measures ANOVA on JNDs revealed no significant main effects or interaction effects (see Table 6). These results indicate transfer of adaptation from the visual to the auditory modality, but not vice versa.

**Table 6:** Repeated-measures ANOVA results on detection thresholds and JNDs assessing transfer effects for adapt-V conditions.

Effects	df	F	*p*	*ƞ*_*p*_^2^
**Detection thresholds**				
Adaptation, test mode	1, 11	0.007	0.94^tt^	< 0.001
Adaptation, test modality	1, 11	1.94	0.19^tt^	0.15
Adaptation delay	1, 11	8.34	0.008*^ot^	0.43
Adaptation, test mode × adaptation, test modality	1, 11	< 0.001	0.98^tt^	< 0.001
Adaptation, test mode × adaptation delay	1, 11	1.27	0.28^tt^	0.10
Adaptation, test modality × adaptation delay	1, 11	1.81	0.21^tt^	0.14
Adaptation, test mode × adaptation, test modality × adaptation delay	1, 11	2.66	0.13^tt^	0.20
**JNDs**				
Adaptation, test mode	1, 11	1.86	0.20^tt^	0.14
Adaptation, test modality	1, 11	0.59	0.46^tt^	0.05
Adaptation delay	1, 11	0.61	0.23^ot^	0.05
Adaptation, test mode × adaptation, test modality	1, 11	0.44	0.52^tt^	0.04
Adaptation, test mode × adaptation delay	1, 11	2.91	0.12^tt^	0.21
Adaptation, test modality × adaptation delay	1, 11	3.25	0.10^tt^	0.23
Adaptation, test mode × adaptation, test modality × adaptation delay	1, 11	0.81	0.39^tt^	0.07

Bold asterisks indicate significant effects. ‘^ot^’ indicate one-sided test values whereas ‘^tt^’ indicate two-sided test values. df, degrees of freedom; F, F value, *p*, *p* value; *ƞ*_*p*_^2^ partial eta-squared.

## Discussion

In the present study, we aimed to disentangle motor-sensory and inter-sensory components of sensorimotor temporal recalibration for visual and auditory feedback. To this end, we presented participants with systematic delays between button presses and sensory feedback (adaptation phase), and tested whether detection of variable delays inserted between action-feedback events changed after adaptation (test phase). To investigate the role of motor-sensory and inter-sensory components, we used active and passive movements at adaptation and test phases. We hypothesized that if predictions based on efference copy signals play a substantial role in the adaptation of sensorimotor events^18–20^, then we would observe limited recalibration for these events when the motor-sensory component was absent at adaptation. Our results indicate that the motor-sensory component contributes more to the temporal recalibration of motor-auditory events than the inter-sensory component, while the inter-sensory component contributes more to the temporal recalibration of motor-visual events than the motor-sensory component. Transfer of recalibration from the visual to the auditory domain, and not from the auditory to the visual domain, highlights modality-specific influences on recalibration. Our results suggest that the extent to which motor-sensory and inter-sensory components contribute to sensorimotor temporal recalibration depends on the modality of the sensory feedback.

Drawing from previous work on sensorimotor recalibration^8,24–26^ and inter-sensory integration^34,36^, we proposed that sensorimotor temporal recalibration results from a temporal remapping of motor-sensory and inter-sensory discrepancies between actions and their sensory feedback, modulated by the sensory feedback modality. Our initial analysis on within-modality conditions revealed a main effect of *adaptation delay*, indicating an overall shift in the detection thresholds between 0 and 150 ms delays across all conditions, and thus overall recalibration. However, post-hoc comparisons on the three-way interaction between *adaptation, test mode*; *adaptation, test modality* and *adaptation delay* suggested that adaptation effects are mainly driven by the adapt-active, test-active conditions, and adapt-passive, test-active condition when feedback modality is visual. Assuming that recalibration in the adapt-passive, test-active condition reflects only the inter-sensory component, the results suggest a differential contribution of the inter-sensory component on sensorimotor temporal recalibration as a function of sensory feedback modality. Apart from threshold differences, we did not find a main effect of adaptation delay on JNDs, suggesting similar temporal integration windows independent of adaptation delays. However, lack of JND differences across adaptation delays does not necessarily mean that other processes such as temporal window of integration do not contribute to sensorimotor temporal recalibration.

The comparison between adaptation across sensorimotor (adapt-active, test-active) and *purely* sensory (adapt-passive, test-passive) events allowed us to examine whether predictions based on efference copy signals provide additional advantages in recalibrating the timing of related events^10,21,38,39^. Our analyses regarding within-modality conditions demonstrate significiant temporal recalibration for motor-auditory and motor-visual events, but not for purely sensory events. Together, these results are in line with the notion of an internal forward model, whereby an efference copy of the motor command predicts the sensory feedback of the action, and provides additional advantage in recalibrating temporal discrepancies between related events^8,14,18–20^.

Our main aim in the present study was to investigate the impact of motor-sensory (corresponding to predictions based on efference copy) and the inter-sensory components (corresponding to reafferent feedback) on sensorimotor temporal recalibration. By introducing a condition with passive button presses at adaptation and active button presses at test, we were able to disentangle the impact of efferent from reafferent feedback, both of which may contribute to recalibration^8,9,23^. First, our analysis on within-modality conditions resulted in a two-way interaction between *adaptation, test mode* and *adaptation, test modality*. Post-hoc comparisons assessing the two-way interaction revealed smaller detection thresholds in the adapt-active, test-active compared to the adapt-passive, test-passive conditions for auditory feedback independent of *adaptation delay*. This indicates higher temporal sensitivity for motor-auditory than for sensory-auditory events, and highlights the impact of motor-sensory component on the temporal perception of motor-auditory events. There is also a trend towards an increase in the detection thresholds from adapt-active, test-active to adapt-passive, test-active, and from adapt-active, test-active to adapt-passive, test-passive conditions when the feedback modality was auditory (see Fig. 2), though this was not significant between adapt-active, test-active and adapt-passive, test-active conditions. Nevertheless, the increasing trend in the thresholds with the exclusion of the motor-sensory component is in line with the role of the motor-sensory component in the timing of motor-auditory events. Second, despite the finding that sensorimotor recalibration for actions with auditory and visual feedback are similar, our results point to the importance of the motor-sensory component for motor-auditory events, that is, when efference copy signals are present^11,17,21,39^. This is evident from the systematic shift in detection thresholds in the adapt-active, test-active condition, but not in the adapt-passive, test-active condition for auditory feedback modality. The lack of a modulatory effect from the inter-sensory component for motor-auditory events might also be explained in terms of audio-tactile interactions in time perception. The timing of audio-tactile events is more susceptible to temporal discrepancies than visuo-tactile events^40,41^, presumably because in the real world such events frequently occur in close physical proximity^42^. This might explain the lack of temporal recalibration between button presses and auditory feedback when only the inter-sensory component was available, and efference copy information was absent. In summary, the absence of adaptation in the adapt-passive, test-active condition for auditory feedback points to the crucial role of the motor-sensory component in the temporal recalibration of motor-auditory events.

Despite an overall advantage of predictions based on efference copy in the temporal recalibration for motor-auditory events, a different pattern emerged for motor-visual events. Recalibration was present for event pairs involving visual modality *independent of* whether predictions based on efference copy were present at adaptation. More specifically, adaptation was demonstrated for adapt-active, test-active as well as for adapt-passive, test-active conditions with visual feedback modality. Our results indicate that temporal adaptation for motor-visual events involves remapping of timing between the inter-sensory component and the visual feedback; that is, adaptation mainly occurs between tactile-proprioceptive feedback from the action and visual feedback of the action^23^. Moreover, lack of significant differences in adaptation between the adapt-active, test-active and adapt-passive, test-active conditions with visual feedback further supports the idea that the inter-sensory component is sufficient to produce recalibration of motor-visual events. Together, these results suggest that, compared to the inter-sensory component, the motor-sensory component contributes more to the recalibration of motor-auditory events, while the inter-sensory component contributes more to the recalibration of motor visual events. This contradicts evidence supporting a supramodal mechanism^6,9^ or the motor-sensory component driving recalibration^8^, and rather indicates differential contributions of motor-sensory and inter-sensory components on sensorimotor temporal recalibration depending on the sensory feedback modality^8,23,27^. In this sense, comparable adaptation in the adapt-passive, test-active and adapt-active, test-active conditions involving visual feedback can be explained by assuming that the motor-sensory component contributes rather weakly to motor-visual recalibration.

In order to better address the mechanism underlying sensorimotor temporal recalibration, we assessed the presence of cross-modal transfer of recalibration. Transfer of recalibration from one modality to another would favor a supramodal mechanism driving recalibration, whereas lack of transfer would indicate modality-specific effects on recalibration. If sensory feedback of the action does *not* influence recalibration, then we should observe transfer of recalibration across modalities. If modality-specific effects are at play, there should be no transfer. The absence of transfer may arise from a shift in the sensory feedback towards the motor-sensory or the inter-sensory component, or both^6,9^. The analysis on detection thresholds assessing transfer of recalibration from the visual to the auditory feedback modality in the adapt-active, test-active and adapt-passive, test-active conditions resulted in a main effect of *adaptation delay*, indicating transfer of adaptation from vision to audition. The transfer from the visual to the auditory domain demonstrates recalibration that is *not* driven by sensory feedback modality. Together with the finding that temporal recalibration for visual events is mostly independent of the motor-sensory component (adaptation in adapt-active, test-active and adapt-passive, test-active conditions for motor-visual events), this suggests that recalibration for motor-visual events is driven mainly by a shift in the inter-sensory component. It should be noted that cross-modal transfer of recalibration might be weaker than recalibration within the same feedback modality (see^6,8^ for transfer effects in the sensorimotor events, and for^43,44^ sensory-sensory events). A similar pattern can be observed in our data (see Fig. 3b, and Fig. 4b). This partial recalibration effect is thought to arise from a cost in switching between different sensory modalities^6^. More evidence is needed to determine the contribution of sensory feedback modality on sensorimotor temporal recalibration.

As discussed above, the inter-sensory component is mainly responsible for the temporal recalibration of motor-visual events. The partial transfer of recalibration from vision to audition in this condition suggests that the tactile-proprioceptive event (i.e., the inter-sensory component rather than the motor-sensory component) is shifted towards the visual feedback. As previous investigations on transfer effects did not involve necessary conditions to disentangle motor-sensory from inter-sensory component^9,27,28^, they could only conclude whether the first or the second event shifts in time. Our experimental design allowed us to disentangle the contributions of motor-sensory and inter-sensory components by the presentation of a passive condition, and provide further evidence that the inter-sensory component can account for sensorimotor temporal recalibration, supporting the findings of Arnold et al.^23^. However, note that in Arnold et al.^23^, the feedback modality was auditory. Our results therefore should be considered with caution, and clearly, further investigation is needed to disentangle the relative contributions of motor-sensory and inter-sensory components to sensorimotor temporal recalibration.

Contrary to the transfer effect observed for motor-visual events, the analysis investigating transfer from audition to vision for adapt-active, test-active conditions indicates a lack of transfer from audition to vision. Together, our results are in line with previous findings showing asymmetric transfer of recalibration for sensorimotor events; that is, transfer of recalibration from the visual to the auditory domain, but not vice versa^27,28^. In this sense, they do not support the notion of a supramodal mechanism being at play for all sensory feedback modalities^6,9^. Instead, our results provide further evidence for the differential contributions of motor-sensory and inter-sensory components as a function of sensory feedback modality.

What might explain the asymmetric transfer effects in our study? At first glance, our results (transfer from vision to audition, but not vice versa) seem in contradiction with the idea of auditory dominance in time perception. If the auditory system encodes temporal information more precisely than the visual system^30–32^, a straightforward expectation would be recalibration transferring from audition to vision. However, our results may highlight the specific involvement of the auditory system in coding time. Indeed, evidence from various imaging studies points to the involvement of the auditory cortex in temporal tasks^45^. Other studies found that the temporal representation of events are mainly encoded in the auditory cortex, independent of the sensory modality^46,47^. The transfer of recalibration from vision to audition in our study can therefore be explained as the auditory system being involved in the recalibration of motor-visual timing, which led to a transfer from vision to audition. However, when the recalibrated modality is auditory, a transfer is unlikely as the auditory system is already involved (see also^27,29^). Another point of consideration is related to which event might have been shifted in time. For motor-visual events, our results support a shift of the inter-sensory component (tactile-proprioceptive feedback) towards visual feedback. For motor-auditory events, the auditory feedback seems to shift towards the motor-sensory component. This might be related to a change in the processing speed of the auditory feedback as a function of delay. Sugano et al.^37^ investigated the existence of a shift in the timing of a sensory feedback associated with an action. Their results suggest that the temporal recalibration of motor-auditory events partly arises from an enhancement (speeding up) in the processing of auditory feedback, which is absent for visual feedback^37^. In our study, the absence of transfer from audition to vision in the adapt-active, test-active condition indicates a shift in the auditory component towards the motor-sensory component. Nevertheless, the possibility of a shift in both directions via the speeding up of auditory processing still exists. We believe this is worth considering, especially in light of evidence on sensorimotor synchronization suggesting a close relationship between motor and auditory systems^48–52^. Behaviorally, the perception of rhythmicity (‘beat’) has been associated more strongly with auditory than visual stimuli^52^. At the neural level, listening to auditory rhythms activate the motor network^48^. In addition, internal representation of rhythm for visual stimuli can be primed with auditory stimuli, but not vice versa, as indicated by the activation in neural structures associated with rhythm and beat perception^50^. Together, these results highlight the close connection between auditory and motor systems, which may modulate temporal recalibration.

The asymmetric transfer of recalibration can be alternatively explained by a change in the reliance on the sensory feedback modality as a result of external factors. Reliance on a sensory modality depends not only on the precision of that modality in encoding the feature of interest (i.e., time), but also on external factors. Because vision travels much faster than sound, especially in the case of a distant event, it might become more dominant than audition in estimating time^53,54^. In fact, Navarra et al.^53^ speculated that because audition cannot be relied on with distal events, there may be a *general* tendency to rely on visual information in the perception of timing. Under this assumption, vision would dominate, attracting the initial (according to our findings, the inter-sensory) component towards itself. Recalibration would then transfer from the visual to the auditory modality. For motor-auditory events, the auditory modality would be attracted towards the initial (according to our findings, motor-sensory) component, and recalibration would not transfer to the visual modality (see also^27^).

To conclude, our results demonstrate that sensorimotor temporal recalibration results from interactions between motor-sensory and inter-sensory components modulated by the sensory feedback modality associated with the action. We suggest that incongruent results on sensorimotor temporal recalibration within and across different sensory modalities can be explained by the differential contributions of motor-sensory and inter-sensory components, which is further modulated by the sensory feedback modality.

## Methods

### Participants

The experiment was approved by the local ethics committee (Ethik-Kommission des Fachbereichs Medizin der Philipps-Universitaet Marburg) and was performed in accordance with the Declaration of Helsinki, except for pre-registration^55^. A total of 14 university students from Philipps University Marburg took part in the study. Data from two participants were discarded from group-level analyses (see “Data analysis” section), resulting in a final sample of 12 participants (seven females, mean age 24.9 ± 1.64). All participants were right-handed as confirmed by the Edinburgh Handedness Inventory^56^. They reported normal or corrected-to-normal vision, and normal hearing. In addition, none reported having current psychiatric or neurological conditions or the use of related medication. Participants provided their written informed consent prior to the experiment, and received monetary compensation for their participation.

### Stimuli and apparatus

Auditory stimuli consisted of brief sine-wave tones (frequency = 2000 Hz, duration =  ~ 33.4 ms with 2 ms rise/fall slopes), and were presented via headphones. Visual stimuli were Gabor patches (2.56°, spatial frequency = 2cycles/degree, duration =  ~ 33.4 ms), and were presented on a 24″ computer monitor (Samsung Syncmaster 2443, 1920 × 1200pixels resolution, 60 Hz frame refresh rate). Stimulus presentation and response recording were controlled by Octave and Psychtoolbox-3^57^. Delay detection responses were recorded via a keyboard (‘V’ and ‘N’ buttons on the keyboard).

The experiment was conducted in a dimly lit room. Participants sat at a desk in front of a monitor with a viewing distance of approximately 55 cm. Their right index finger was placed on a custom-made button. The custom-made electromagnetic button was used to trigger auditory and visual stimuli. Stroke length of the button was 5 mm with a light-barrier triggered within the last 0.2 mm of movement. Both voluntary manual and externally activated button presses were recorded by the computer as a left click of a USB mouse. Therefore, jitter and delay of the button press did not depend on whether the movement was active or passive. To ensure that the index finger was pulled by the button for passive movements, cotton bandages were used to fix the finger to the button. The cotton bandages were used during the execution of active movements as well. For active button presses, the initial force was 1.5 Newton (N), as measured by a spring force gauge, slowly increasing to approximately 2.5 N in the final position. For passive button presses, the finger was initially pulled with approximately 1 N, and the force increased to approximately 4 N in the final position. The duration of the button press for the passive movement was set to 300 ms based on previous studies^12,41^. For both movements, auditory or visual stimuli were presented only when the button was pressed down completely. In addition, a cushion was provided to ensure a comfortable hand/forearm positioning. The button pad was covered with a black box to prevent the participant from using visual cues from their hand or finger to perform the delay detection task. White noise was presented throughout the experiment to mask any auditory cues, especially the mechanical sound from the passively pressed button. Earplugs were worn by the participants to attenuate any external sound. Prior to the experimental blocks, each participant was asked whether they could hear the tones clearly with the ear plugs and the additional white noise.

### Design

**Figure 5:**
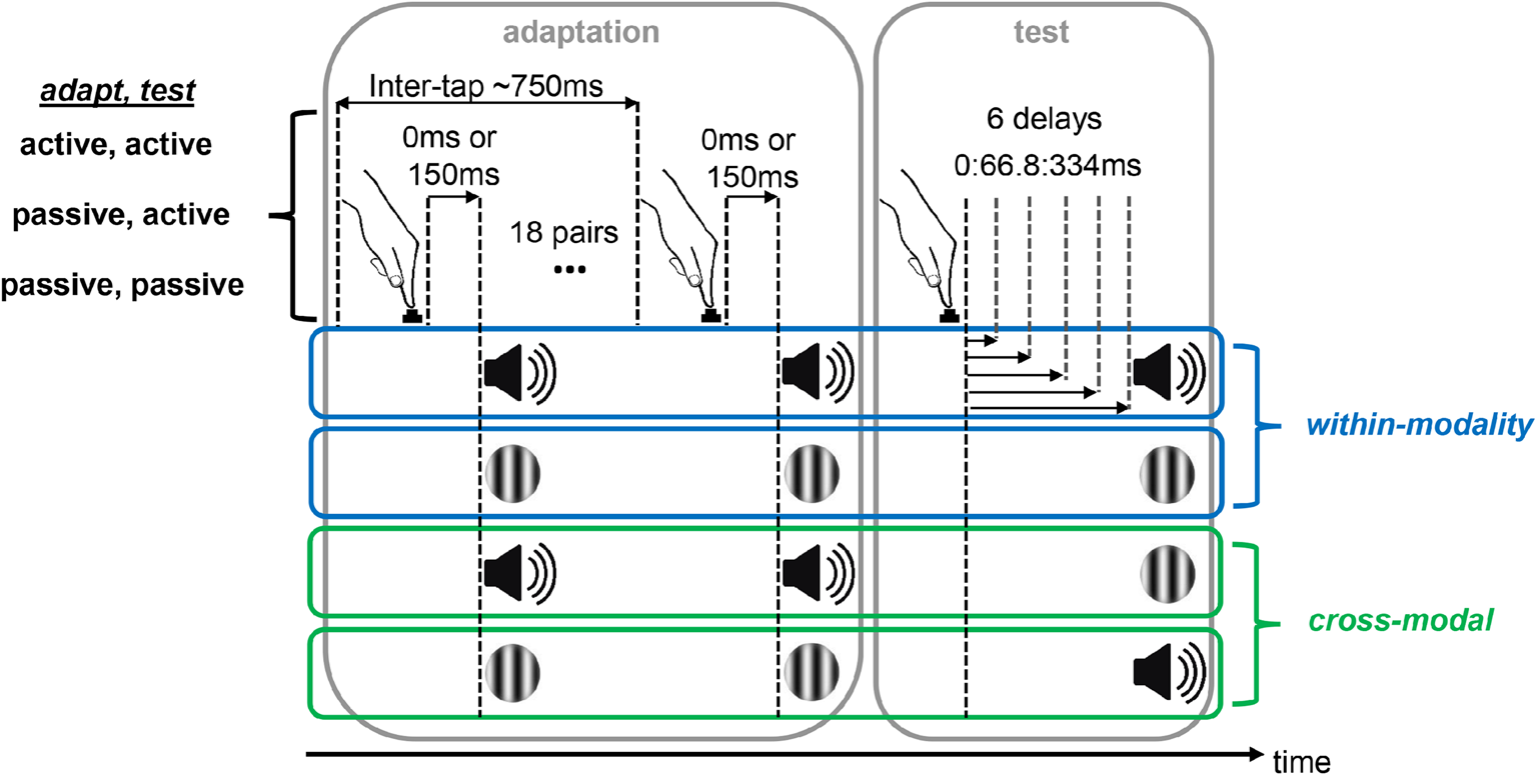
Schematic of the experimental conditions. In the adaptation phase, participants adapted to a systematic delay of either 0 ms or 150 ms between button press and sensory feedback. Inter-tap interval between the button press and the sensory feedback was ~ 750 ms. In the test phase, the sensory feedback was presented with variable delays (0–334 ms), and participants were asked to report whether they detected such a delay.

A schematic of the experimental conditions is outlined in Fig. 5. The experimental design involved four within-subjects factors. The first factor corresponded to the *adaptation, test mode*, defining the action to be performed in the adaptation and test phases. The action could either be active in which the participant pressed the button, or passive in which the button was depressed automatically without the participant pressing it down. Actions in the *adaptation, test mode* pairs could be adapt-active, test-active (sensorimotor recalibration), adapt-passive, test-passive (sensory-sensory recalibration), or adapt-passive, test-active (inter-sensory component in sensorimotor recalibration). The second factor concerned the modality of sensory feedback in the adaptation phase (*adaptation modality*) that could either be auditory (adapt-A) or visual (adapt-V). The third factor corresponded to the modality of sensory feedback during test (*test modality*), that could be either within-modality test (adapt-A, test-A; adapt-V, test-V) or cross-modal test (adapt-A, test-V; adapt-V, test-A). Finally, the fourth factor was *adaptation delay*, defining the time between the action and the sensory feedback at adaptation. Effects of this factor indicate whether adaptation took place. When *adaptation delay* was 150 ms, we expected a decrease in the perceived delay between action and feedback in the test phase, compared to when it was 0 ms. The dependent variable was delay detection judgments from six delays (0, 66.8, 133.6, 200.4, 267.2, 334 ms) inserted between the active or the passive button press, and the auditory or visual feedback at the test phase.

Participants attended eight experimental sessions completed on separate days. In each session, the participants completed six blocks of different experimental conditions. Levels of the factor *adaptation delay* were presented on separate days in order to prevent possible carryover effects between adaptation delays^9,27^. Moreover, to prevent fatigue, we further limited the number of conditions received within a day to six by presenting levels of the factor *adaptation modality* on separate days. Levels of factors *adaptation, test mode* and *test modality* were presented on the same day in a pseudorandomized order. We split the experimental conditions in half, so that the first and last four sessions consisted of the same conditions. This was necessary to have a consistent estimate of the detection responses per condition without exhausting the participants.

### Procedure

Each block consisted of 18 trials, all of which involved an adaptation phase and a test phase. On each trial, there were 18 button press-sensory feedback events at adaptation, and six button press-sensory feedback pairs with six variable delays at the test phase. Delays at the test phase were presented in a pseudorandomized order so that each delay occurred equally often at each presentation order. The number of repetitions in the adaptation phase and the delays in the test phase were determined based on two previous pilot studies. In the adaptation phase, the participants were asked to perform button presses at a constant pace. Each button press would lead to the occurrence of an brief tone or a Gabor patch. For trials in which the participant would actively initiate button presses (adapt-active), they were required to press the button approximately every 750 ms. For trials in which the button was pressed automatically (adapt-passive), they were asked to let the button go down, and not exert any force on the button. In the test phase, the button presses could either be active (test-active) or passive (test-passive), this time, leading to the presentation of a tone or a Gabor patch with variable delays. The participants were asked to detect a delay between the button press and the tone or the Gabor patch.

**Figure 6:**
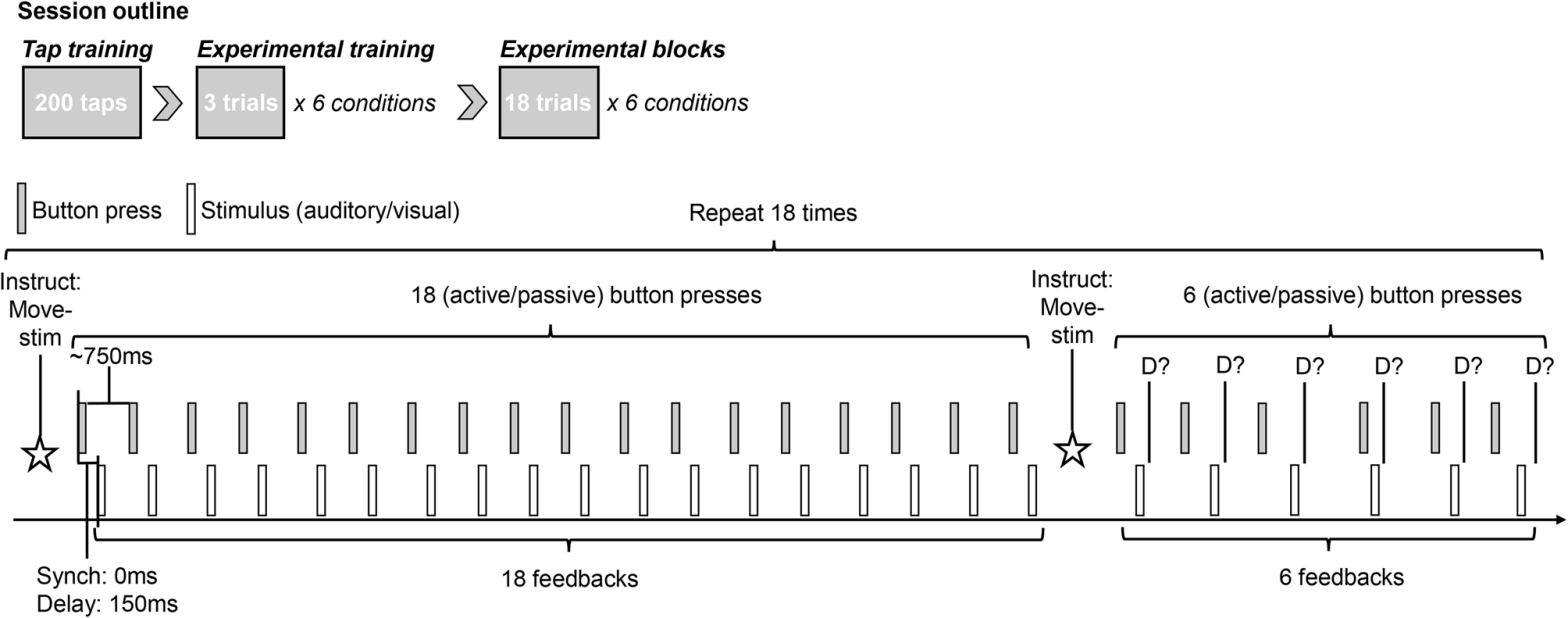
Outline of an experimental session (top). Each session consisted of a tap training block and six experimental training blocks, followed by six experimental blocks. Timeline of an experimental trial (bottom). Each trial consisted of an adaptation phase and a test phase, each preceded by instructions (*instruct*) on the movement type (*move*: active or passive) and sensory feedback received (*stim*: auditory or visual). Adaptation phase consisted of 18 button press-feedback pairs, with (150 ms) or without (0 ms) a systematic delay between the two. Test phase consisted of six button press-feedback pairs, each of which involved variable delays (0–334 ms) in between. Immediately after the button press-feedback pair, the participants were asked to judge whether there was a delay between these two events (*D?*).

A schematic of an experimental trial is shown in Fig. 6. Each block began with instruction of the movement type (button press: active or passive) as well as the modality at adaptation (auditory or visual) for 1500 ms. During this time a fixation cross was presented, which remained on the screen for 300 ms after the instruction. The fixation cross then disappeared, prompting participants to perform button presses at the instructed pace actively or passively by letting the finger press the button. Each button press triggered a tone or a Gabor patch on the screen, either immediately (0 ms delay) or delayed in time by 150 ms. After the completion of 18 button presses, an instruction followed for 1500 ms, informing the participant about movement type and sensory modality in the upcoming test phase (active/passive auditory/visual). The fixation cross disappeared again, prompting the participant to press the button, this time, once. Each button press triggered a tone or a Gabor patch with one of the following six delays: 0, 66.8, 133.6, 200.4, 267.2, and 334 ms. A 500 ms interval followed in which the fixation cross appeared again. After this interval, the question ‘Delay?’ was presented on the screen. The participants used the keys ‘V’ and ‘N’ for ‘Yes’ and ‘No’ to provide their responses. They had to register their responses within 2000 ms. The assignment of keys to yes/no responses was counterbalanced across participants. Six test trials were presented at each test phase of a block, with all trials having one of the six delays. The order of delay presentation was pseudorandomized within a block so that all delays appeared equally often at each position in the trial. The test phase was followed by an inter-trial interval ranging from 500 to 1500 ms. For trials in which the button was actively pressed at adaptation, participants were informed how to adjust their pace of button pressing on the next trial. If the overall (median) interval between button presses fell within the range of 600–900 ms, a ‘keep the pace’ instruction appeared for 1500 ms after the test phase and prior to the inter-trial interval. If the median intervals were below 600 ms and above 900 ms, participants received ‘Slower’ and ‘Faster’ instructions, respectively. These *slow* or *fast* trials were immediately repeated until the median button press interval was within the expected range. Overall, most participants were able to keep the pace (2.8% of all trials had to be repeated).

Prior to the experimental blocks, participants practiced tapping in synchrony with a metronome for 3 min in order to familiarize themselves with the correct pace of the button presses. The participants were also asked whether they felt comfortable with their performance, and if not, were offered further practice. All participants were able to tap in accordance with the auditory signal in the initial training. The tapping practice was followed by short training blocks of the experimental conditions. These training blocks consisted of three trials, and were performed before the first experimental block of a session. We encouraged participants to take breaks between the blocks, but did not force a fixed break. Each trial lasted for ~ 30 s, and each experimental block for ~ 9 min. The duration of the entire experiment over the eight sessions was approximately 10.5 h.

### Data analysis

The data were subjected to a two-stage inspection procedure. First, we identified extreme detection responses for each participant separately. Second, we checked the overall distribution of detection responses for each condition to determine the appropriate statistical analyses.

In order to discover extreme response patterns, we plotted the proportion of detection responses (i.e., ‘Yes’ responses) as a function of the action-feedback delay at test for each participant and condition. We inspected the detection responses in terms of proportion of detection at the smallest and largest delay trials. For this, we pooled the data across adaptation delays (0 and 150 ms) and modalities (within and cross-modal) per recalibration mode. We then calculated the median of proportion detected responses for the largest delay. Data from two participants was excluded from further analyses because their median of proportion detected responses exceeded 50% at no delay trials (suggestive of a strategy to press 'yes', even when there was no delay) or did not exceed 50% at the largest delays (chance-level detection). The remaining data from each participant and condition were fitted to a cumulative Gaussian using psignifit 4.0^58^ and Matlab 2019a^59^. This version of psignifit adopts Bayesian inference to estimate detection parameters^58^. From fitting psychometric functions, we obtained estimates of detection thresholds (50% point of the psychometric function), and JNDs (difference between the 50% and 84% points of the psychometric function). These estimates were used to conduct statistical analyses. Mauchly's Test of Sphericity indicated that the assumption of sphericity had not been violated. We performed repeated measures ANOVAs for comparisons across conditions. For each *adaptation, test mode* and *modality* pairing, recalibration was defined as a systematic change in the perception of time as a function of the temporal delay presented at the adaptation phase between the action and the sensory feedback; this would correspond to a change in the detection thresholds or increase in JNDs across delays.

In order to assess the existence of recalibration for different events, and the contribution of motor-sensory and inter-sensory components, we conducted a 3 (*adaptation, test mode*: adapt-active, test-active vs. adapt-passive, test-active vs. adapt-passive, test-passive) × 2 (*adaptation, test modality*: adapt-A, test-A vs. adapt-V, test-V) × 2 (*adaptation delay*: 0 ms vs. 150 ms) repeated measures ANOVA on thresholds and JNDs.

Results of the first analysis revealed modality-specific effects on sensorimotor temporal recalibration (see “Results” section). We therefore assessed cross-modal transfer separately for each adaptation modality. In addition, as we were interested in transfer effects across modalities, we took into account only those conditions in which we found significant within-modality recalibration. Accordingly, we conducted a 2 (*adaptation, test modality*: adapt-A, test-A vs. adapt-A, test-V) × 2 (*adaptation delay*: 0 ms vs. 150 ms) repeated measures ANOVA on thresholds and JNDs for the adapt-A condition, and a 2 (*adaptation, test mode*: adapt-active, test-active vs. adapt-passive, test-active) × 2 (*adaptation, test modality*: adapt-V, test-V vs. adapt-V, test-A) × 2 (*adaptation delay*: 0 ms vs. 150 ms) repeated measures ANOVA on thresholds and JNDs for the adapt-V condition. Nevertheless, significant cross-modal recalibration may be observed in the absence of within-modality recalibration. This might arise from different factors such as task performance in the test modality or sensitivity of the task itself. We therefore provided the unrestricted analyses (including all adaptation, test modalities even when significant within-modality recalibration is lacking) in the Supplementary Materials (see Supplementary Materials).

Inferential statistics were computed with frequentist hypothesis tests (α = 0.05). Due to our specific hypothesis regarding *adaptation delay* (increased thresholds for 150 ms compared to 0 ms in a specific condition), we carried out directed (one-tailed) tests for the main effect of *adaptation delay*. For all other main or interaction effects (involving *adaptation, test mode* and *adaptation, test modality*), we carried two-tailed tests. For interaction effects, planned post-hoc comparisons were performed when multiple comparisons were made, and Bonferroni correction was applied when necessary.

## Supplementary Information

Supplementary Information.

## Data Availability

The data will be provided online at the pre-registered doi: osf.io/c27 × 6/, and can be used for non-commercial research purposes upon acceptance of this article for publication.
