# Supplementary material for: Different contributions of efferent and reafferent feedback to sensorimotor temporal recalibration
Source: Sci Rep. 2021 Nov 19;11:22631. doi: 10.1038/s41598-021-02016-5 (PMC8604902; doi:10.1038/s41598-021-02016-5)
Supplement: Supplementary file 1 — Supplementary Information. [file 41598_2021_2016_MOESM1_ESM.docx]

**Supplementary Materials**

**Cross-modal temporal recalibration**

We assessed the existence of cross-modal temporal recalibration separately for each adaptation modality. For this, we conducted a 3 (*adaptation, test mode*: adapt-active, test-active vs. adapt-passive, test-active vs. adapt-passive, test-passive) x 2 (*adaptation delay*: 0ms vs. 150ms) analysis on thresholds and JNDs separately for adapt-A, and adapt-V conditions.

The 3 (adaptation, test mode: adapt-active, test-active vs. adapt-passive, test-active vs. adapt-passive, test-passive) x 2 (adaptation delay: 0ms vs. 150ms) repeated measures ANOVA on JNDs for adapt-A, test-V revealed no significant main or interaction effects (see Table 1).

The 3 (adaptation, test mode: adapt-active, test-active vs. adapt-passive, test-active vs. adapt-passive, test-passive) x 2 (adaptation delay: 0ms vs. 150ms) repeated measures ANOVA on thresholds for adapt-V, test-A resulted in a main effect of adaptation delay (see Table 2). The 3 (adaptation, test mode: adapt-active, test-active vs. adapt-passive, test-active vs. adapt-passive, test-passive) x 2 (adaptation delay: 0ms vs. 150ms) repeated measures ANOVA on JNDs revealed no significant main or interaction effects (see Table 2).

| **Detection thresholds**  **Effects** | **df** | **F** | **p** | ***ƞ_p_^2^*** |
| --- | --- | --- | --- | --- |
| *adaptation, test mode* | 2, 22 | .44 | .65^tt^ | .04 |
| *adaptation delay* | 1, 11 | 2.73 | .06^ot^ | .20 |
| *adaptation, test mode* x *adaptation delay* | 2, 22 | .02 | .98^tt^ | <.01 |
|  |  |  |  |  |
| **JNDs**  **Effects** | **df** | **F** | **p** | ***ƞ_p_^2^*** |
| *adaptation, test mode* | 2, 22 | 1.38 | .27^tt^ | .11 |
| *adaptation delay* | 1, 11 | 1.16 | .15^ot^ | .10 |
| *adaptation, test mode* x *adaptation delay* | 2, 22 | .60 | .56^tt^ | .05 |

**Table 1.** Repeated-measures ANOVA results on detection thresholds and JNDs for adapt-A, test-V conditions. ‘^ot^’ indicate one-tailed test values whereas ‘^tt^’ indicate two-tailed test values. df, degrees of freedom; F, F value, p, p value; ***ƞ_p_^2^*** partial eta-squared.

| **Detection thresholds**  **Effects** | **df** | **F** | **p** | ***ƞ_p_^2^*** |
| --- | --- | --- | --- | --- |
| *adaptation, test mode* | 2, 22 | 2.96 | .07^tt^ | .21 |
| *adaptation delay* | 1, 11 | 5.12 | .02*******^ot^ | .32 |
| *adaptation, test mode* x *adaptation delay* | 2, 22 | 1.23 | .31^tt^ | .10 |
|  |  |  |  |  |
| **JNDs**  **Effects** | **df** | **F** | **p** | ***ƞ_p_^2^*** |
| *adaptation, test mode* | 2, 22 | .33 | .72^tt^ | .03 |
| *adaptation delay* | 1, 11 | .57 | .23 ^ot^ | .05 |
| *adaptation, test mode* x *adaptation delay* | 2, 22 | 1.70 | .21^tt^ | .13 |

**Table 2.** Repeated-measures ANOVA results on detection thresholds and JNDs for adapt-V, test-A conditions. Bold asterisks indicate significant effects. ‘^ot^’ indicate one-sided test values whereas ‘^tt^’ indicate two-sided test values. df, degrees of freedom; F, F value, p, p value; ***ƞ_p_^2^*** partial eta-squared.
